# Serum vascular endothelial growth factor b and metabolic syndrome incidence in the population based cohort Di@bet.es study

**DOI:** 10.1038/s41366-022-01212-1

**Published:** 2022-08-20

**Authors:** Ana Lago-Sampedro, Said Lhamyani, Sergio Valdés, Natalia Colomo, Cristina Maldonado-Araque, Inmaculada González-Molero, Viyey Doulatram-Gamgaram, Elias Delgado, Felipe J. Chaves, Luis Castaño, Alfonso Calle-Pascual, Josep Franch-Nadal, Gemma Rojo-Martínez, Sara García-Serrano, Eva García-Escobar

**Affiliations:** 1 CIBERDEM, ISCIII, Madrid, Spain; 2 UGC Endocrinología y Nutrición. Hospital Regional Universitario de Málaga. IBIMA Plataforma BIONAND, Málaga, Spain; 3 Departamento de Medicina y Dermatología, Universidad de Málaga-UMA, Malaga, Spain; 4 CIBEROBN, ISCIII, Madrid, Spain; 5 CIBERER, ISCIII, Madrid, Spain; 6 Department of Endocrinology and Nutrition, Central University Hospital of Asturias/University of Oviedo, Health Research Institute of the Principality of Asturias (ISPA), Oviedo, Spain; 7 Genomic and Genetic Diagnosis Unit, Research Foundation of Valencia University Clinical Hospital-INCLIVA, Valencia, Spain; 8 Cruces University Hospital, BioCruces Bizkaia, UPV/EHU, Endo-ERN, Barakaldo, Spain; 9 Department of Endocrinology and Nutrition, San Carlos University Hospital of Madrid, Madrid, Spain; 10 EAP Raval Sud, Catalan Institute of Health, GEDAPS Network, Primary Care, Research support unit (IDIAP—Jordi Gol Foundation), Barcelona, Spain

**Keywords:** Metabolic syndrome, Metabolic syndrome

## Abstract

**Background/Objectives:**

Although vascular endothelial growth factor b (VEGFb) might have an impact on the development of obesity, diabetes and related disorders, the possible relationship between VEGFb serum levels and the incidence of these metabolic complications in humans is still unknown. The aim of our study was to evaluate the association between VEGFb serum levels and the new-onset of metabolic syndrome (MS) and its components in the Spanish adult population after 7.5 years of follow-up.

**Subjects/Methods:**

A total of 908 subjects from the Di@bet.es cohort study without MS at cross-sectional stage according to International Diabetes Federation (IDF) or Adult Treatment Panel III (ATP-III) criteria were included. Additionally, five sub-populations were grouped according to the absence of each MS component at baseline. Socio-demographic, anthropometric and clinical data were recorded. The Short Form of International Physical Activity Questionnaire (SF-IPAQ) was used to estimate physical activity. A fasting blood extraction and an oral glucose tolerance test were performed. Serum determinations of glucose, lipids, hsCRP and insulin were made. VEGFb levels were determined and categorized according to the 75th percentile of the variable. New cases of MS and its components were defined according to ATPIII and IDF criteria.

**Results:**

A total of 181 or 146 people developed MS defined by IDF or ATP-III criteria respectively. Serum triglyceride levels, hs-CRP and systolic blood pressure at the baseline study were significantly different according to the VEGFb categories. Adjusted logistic regression analysis showed that the likelihood of developing MS and abdominal obesity was statistically reduced in subjects included in the higher VEGFb category.

**Conclusion:**

Low serum levels of VEGFb may be considered as early indicators of incident MS and abdominal obesity in the Spanish adult population free of MS, independently of other important predictor variables.

## Introduction

The metabolic syndrome (MS) can be defined as a cluster of metabolic dysfunctions which is characterized by the increase in abdominal obesity, insulin resistance, hypertension and hyperlipidemia coexisting in the same individual [1]. MS is the result of the interaction of genetic and environmental factors, where lifestyle plays an important role in its development. A healthy lifestyle based on adequate nutrition, regular physical activity and control of overweight and obesity are the general recommendations for the treatment and prevention of MS [2]. Among the different causes of MS, physical activity is a fundamental part of its understanding since a sedentary lifestyle has been associated with weight gain and an increase in visceral fat, favouring the appearance of other risk factors such as arterial hypertension, hyperglycemia and dyslipidemia [3]. A chronic, low magnitude inflammatory state has also been related in many studies to the presence of the components of metabolic syndrome, which can be explained in part by the alterations of the adipose tissue and that can be compensated with adequate physical activity [4, 5].

Various diagnostic criteria have been proposed to define MS, but the most internationally accepted ones are from the International Diabetic Federation (IDF) [6] and the National Cholesterol Education Program Adult Treatment Panel III (ATP-III) [7]. The main differences between these definitions are that IDF considers abdominal obesity as a pre-requisite in the diagnosis of MS and the thresholds used for abdominal obesity criteria are different (IDF recommended a cut off for waist circumference of 94 cm for men and 80 cm for women in Europeans, while ATP-III recommends cut-off points of 102 and 88 cm, respectively). Both the prevalence and the incidence of MS vary based on the criteria used to define it.

Several epidemiological and clinical studies have confirmed that the presence of MS directly increases the risk of cardiovascular diseases, diabetes and all causes of mortality [5, 8–10], therefore, novel strategies to detect, protect and prevent future MS development are needed.

A large body of evidence suggests that an altered crosstalk between adipocytes and endothelial cells might be underling the pathogenesis of obesity and consequent metabolic disturbances included in the definition of MS [11–13]. Communication between adipocytes and endothelial cells mostly takes place through the vascular endothelial growth factors and their receptors [11–13]. Among these endothelial growth factors, the endothelial growth factor b (VEGFb) is a member which is abundantly expressed in metabolically active tissues that has been suggested to have an important role regulating the pathogenesis of insulin resistance and which is associated with metabolic disturbances [14–17].

The association between VEGFb serum levels and the pathological lipid accumulation associated with MS or its components has previously been studied in both preclinical and clinical studies [15, 16]. While most preclinical studies have suggested a dual role of VEGFb in the pathogenesis of these metabolic disturbances, via the regulation of endothelial fatty acid uptake and extra-adipose lipid accumulation [18–20] and via the modulation of tissue vascularity [21, 22], its possible role as a biomarker of disease in humans is still unknown. Several authors have reported direct associations between serum or plasma VEGFb levels and some MS components [23–26] that have not been fully replicated in other studies [27, 28]. All these previous clinical investigations were focused on disentangling the association of VEGFb with the presence of MS or its components; however, until now, there are no data regarding the possible relationship between VEGFb levels with the future development of these metabolic complications in humans.

To study blood levels of this growth factor in subjects without metabolic syndrome and their association with the new onset of metabolic disturbances, together with its interrelation with other important explanatory variables, such as physical activity or the underlying inflammatory state of people, may represent a first step towards a better understanding of the complex role of VEGFb in human physiology. Therefore, in this study, we intended to investigate the association between serum VEGFb levels and the new-onset of MS and its components in a Spanish adult population after 7.5 years of follow-up.

## Material/subjects and methods

### Study design, setting and population

**Fig. 1:**
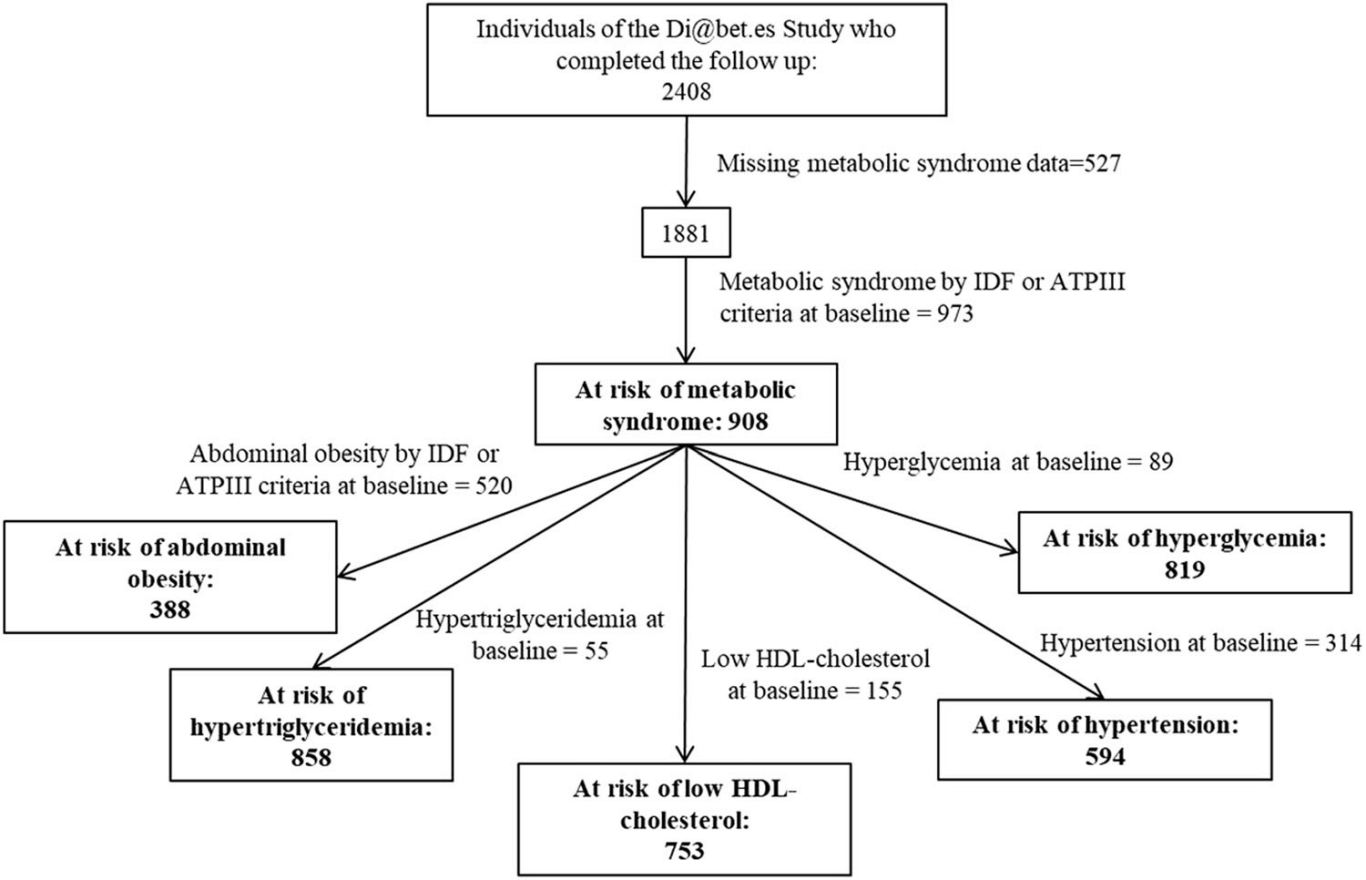
Flow diagram of the cohort study. International Diabetes Federation, ATP-III National Cholesterol Education Program Adult Treatment Panel III, HDL high density lipoprotein.

Samples and data were based on the population-based, cohort study Di@bet.es epidemiological trial (NCT02542735). The Di@bet.es study is a cohort of 5072 subjects older than 18 years old, randomly selected from National Health System registries in 2008–10; this sample is representative of the non-institutionalized Spanish population. Detailed information on the methodology of the Di@bet.es cohort study has been previously described [29, 30]. The Di@bet.es cohort was re-evaluated in 2016-17 (the follow-up time was 7.5 ± 0.6 years) and finally 2408 subjects completed the follow-up [30]. For the present analysis, a data set consisting of 1881 subjects with no missing data about MS has been used, of which 908 were free of MS according to IDF and ATP-III definition criteria in the cross-sectional study (global population at risk). Additionally, five sub-populations were grouped according to the absence of each different MS component at baseline, in order to study the association of VEGFb levels and the development of the MS components. A flow diagram of the study is presented in Fig. 1.

The research was carried out in accordance with the Declaration of Helsinki (WHO 2011) of the World Medical Association. The study was approved by the Ethics and Clinical Investigation Committee of the Hospital Regional Universitario de Málaga (Malaga, Spain) in addition to other regional ethics and clinical investigation committees all over Spain. Written informed consent was obtained from all the participants.

### Data collection and laboratory measurements

In both phases of the study, the participants were invited to attend an examination visit at their health center with a nurse specially trained for this project. Information was collected using an interviewer administered structured questionnaire, followed by a physical examination and blood sampling.

For the present study, the anthropometric variables age, sex, weight and waist circumference were considered. To evaluate the physical activity of the participants, the Short Form of International Physical Activity Questionnaire (SF-IPAQ) was administered [31]. According to the SF-IPAQ score, each individual was assigned to one of three categories: high, moderate and low physical activity as previously reported [29]. Also, clinical variables such as blood pressure levels, fasting levels of glucose, insulin and lipid profile (total cholesterol, high-density lipoprotein (HDL), low-density lipoprotein (LDL) and triglycerides), measured by standard methods, were considered. As an estimator of the subclinical inflammatory state of the subjects, the serum levels of high-sensitivity C-Reactive Protein (hs-CRP) measured with an ultra-sensitive method were used (immunochemiluminescence on an Architect I8000 Analyzer [Abbott Laboratories SA, Madrid, Spain]). Body mass index (BMI) and the homeostasis model assessment (HOMA) were calculated [32].

### Determination of VEGFb levels

Serum VEGFb levels were measured by a commercial human VEGFb ELISA kit (Cloud-Clon Corp, Wuhan, China) in accordance with the manufacturer’s protocol. The detection range of the VEGFb ELISA test was 15.6 to 1000 pg/mL, and intra-assay and inter-assay variations were <10% and <12%, respectively. The detection limit of this ELISA kit is 5.5 pg/mL. VEGFb levels were also categorized according to the 75th percentile of the variable measured in the population at risk of MS (*n* = 908).

### Definition of new cases of MS and its components

We diagnosed new cases of MS according to the IDF or ATP-III criteria. The ATP-III criteria [7] require the presence of at least three of the following components: abdominal obesity (waist circumference ≥102 cm for European men or ≥88 cm for European women), hypertriglyceridemia (triglycerides ≥ 150 mg/dL or receiving lipid lowering drugs), low HDL cholesterol (HDL cholesterol ≤40 mg/dL for men or 50 mg/dL for women or receiving drug treatment), hypertension (systolic/diastolic blood pressure ≥130/85 mmHg or receiving drug treatment) and hyperglycemia (fasting glucose ≥100 mg/dL or receiving antidiabetic drugs). The IDF criteria of MS [6] uses central obesity (waist circumference ≥94 cm for European men or ≥80 cm for European women. If BMI is >30 kg/m², central obesity can be assumed and waist circumference does not need to be measured) as a mandatory criterion together with the presence of at least two of the other four criteria which are identical to those provided by ATP-III.

New cases of every component of the MS definition were diagnosed within the sub-population group at risk of this specific component, according to the criteria described in the paragraph above. New cases of abdominal obesity were diagnosed according to IDF or ATP-III criteria.

### Statistical analysis

Unless otherwise specified, data are presented as means ± standard deviation, or proportions with confidence interval. Differences in baseline variables according to VEGFb categories (defined according to the 75th percentile of the distribution of VEGFb in the population free of MS) were analysed by generalized linear models adjusted by sex, age and BMI or Chi-square test. Variables not following a normal distribution were log-transformed to achieve normalization. Levene’s test has been used to study the homogeneity of the variances, and if it is significant, variance stabilizing transformations (generally logarithm) have been used to carry out the hypothesis contrast tests. Odds ratios for the development of MS and its components according to the VEGFb categories were calculated using logistic regression models adjusted by potential confounders such as age, sex, fasting glucose, waist circumference, serum lipids, hs-CRP, blood pressure and physical activity. The test of Hosmer and Lemeshow was used to check the goodness of ft. For incidence and odds ratios, 95% confidence intervals were computed. Low VEGFb category was used as the reference category in the multivariate logistic regression models.

## Results

### Baseline characteristics of the population according to the VEGFb categories

The study sample included 908 individuals with a mean age of 45 years (age range 18–87 years) from which 36.9% were men. VEGFb levels ranged between 2.75 and 179 mg/dl (median of 40.86 mg/dl). The 75th percentile for VEGFb distribution was 59.74 mg/dl, which is the value used to define the categories of low and high VEGFb.

Differences in baseline characteristics according to these VEGFb categories are presented in Table 1 adjusted by sex, age and BMI as appropriate. Subjects with VEGFb levels over the 75^th^ percentile of the variable (High_VEGFb) were older and with a higher percentage of men than those with VEGFb levels under the 75^th^ percentile of the variable (Low_VEGFb). Serum triglyceride and hs-CRP levels as well as systolic blood pressure were significantly different according to the VEGFb categories, being higher in subjects in the High_VEGFb category. Physical activity was also different according to the VEGFb categories. There were no significant differences in the other variables according to the VEGFb categories.

**Table 1:** General clinical characteristics according to the VEGFb categories.

	Low_VEGFb (*n* = 681)	High_VEGFb (*n* = 227)	*p* value*
Age (years)	43.65 ± 13.33	49.23 ± 14.34	<0.001
Sex (% men)	34.8	43.2	0.02
BMI (Kg/m2)	26.11 ± 4.10	26.21 ± 3.74	0.11
Waist circumference (cm)	87.68 ± 12.16	89.49 ± 11.61	0.54
Fasting glucose (mg/dl)	88.80 ± 12.04	89.20 ± 13.11	0.53
Fasting insulin (mU/dl)	7.14 ± 4.59	7.12 ± 3.71	0.65
HOMA index	1.38 (0.96–1.91)	1.43 (1.02–1.87)	0.81
Total cholesterol (mg/dl)	193.85 ± 35.20	200.38 ± 36.16	0.39
HDL cholesterol (mg/dl)	55.67 ± 11.95	54.88 ± 12.24	0.30
LDL cholesterol (mg/dl)	103.54 ± 29.21	107.79 ± 27.62	0.58
Triacylglycerides (mg/dl)	84.1 (64.7–108.9)	91.2 (73.7–116.0)	0.008
Systolic blood pressure (mmHg)	123.25 ± 16.70	123.49 ± 16.25	0.004
Diastolic blood pressure (mmHg)	73.29 ± 9.38	73.69 ± 8.94	0.172
hs-CRP	1.1 (0.57–3.4)	1.4 (0.7–3.1)	0.008
SF-IPAQ score			0.005
Low %(CI%)	42.2 (38.5–45.9)	34.1 (27.9–40.3)	
Moderate %(CI%)	36.0 (32.4–39.6)	33.6 (27.5–39.7)	
High %(CI%)	21.8 (18.7–24.9)	32.3 (26.2–38.4)	

Data presented as Mean ± standard deviation, median (25th–75th percentile) or proportions(confidence interval). *Differences according to the VEGFb categories measured by univariant generalized linear model adjusted by sex, age and BMI or Chi-Square test.

*VEGFb* vascular endothelial growth factor b, *BMI* body mass index, *HDL* high-density lipoprotein, *LDL* low-density lipoprotein, *HOMA* homeostasis model assessment, *hs-CRP* high-sensitivity C-Reactive Protein, *SF-IPAQ score* Short Form of International Physical Activity Questionnaire Score.

### Metabolic syndrome incidence

In the follow-up, 181 subjects developed MS defined by IDF criteria after 7.5 years, while 146 were diagnosed with MS according to the ATP-III criteria (Table 2). The proportion of subjects who had developed MS by ATP-III criteria was lower in subjects in the High_VEGFb category, however this difference did not reach significance when IDF criteria for MS diagnosis was applied (Table 2).

**Table 2:** Cumulative incidence of metabolic syndrome in all population and stratified by physical activity according the VEGFb categories.

	All population			Low VEGFb group	High VEGFb group	
	N at risk	New cases	Incidence (%(CI%))	Incidence (%(CI%))	Incidence (%(CI%))	*p* value*
IDF	908	181	19,9 (17.3–22.5)	0,20,7 (18.1–23.3)	0,17,6 (15.1–20.1)	0.33
ATP-III	908	146	16,1 (13.7–18.5)	0,17,76 (15.3–20.2)	0,11,01 (9.0–13.0)	0.01
Low physical activity						
IDF	365	82	22,5 (18.2–26.8)	21,6 (17.4–25.8)	26,0 (21.5–30.5)	0.2
ATP-III	365	64	17,5 (13.6–21.4)	16,7 (12.9–20.5)	20,8 (16.6–25.0)	0.2
Moderate physical activity						
IDF	322	65	20,2 (15.8–24.6)	20,8 (16.4–25.2)	18,4 (14.2–22.6)	0.4
ATP-III	322	54	16,8 (12.7–20.9)	19,6 (15.3–23.9)	7,9 (7.4–8,3.4)	0.01
High physical activity						
IDF	221	73	15,4 (10.6–20.2)	18,9 (13.7–24.1)	8,2 (4.6–11.8)	0.03
ATP-III	221	28	12,7 (8.3–17.1)	16,9 (12.0–21.8)	4,1 (1.5–6.7)	0.004

^*^p value for the differences according to VEGFb categories measured by Chi-Square test.

*CI* confidence interval, *VEGFb* vascular endothelial growth factor b, *IDF* International Diabetes Federation, *ATP-III* National Cholesterol Education Program Adult Treatment Panel III.

We have also analyzed the incidence of MS stratifying by physical activity. We found that in sedentary people the incidence of MS was higher in subjects with High_VEGFb, although the difference did not reach significance for any of the criteria. However, people with moderate or high physical activity presented a lower incidence of MS if they had high levels of VEGFb (Table 2).

The results from the multivariate analysis for the development of MS at 7.5 years of follow-up showed that, for both IDF and ATP-III definition criteria, the presence of the High_VEGFb category was an independent protecting factor for the development of MS. The effect was highly significant in the case of diagnosing MS by the ATPIII criterion (Table 3), both in the minimally adjusted model (Model 1) and in the fully adjusted ones where hs-CRP and physical activity were included as covariates. Model 4 also included an interaction term between the categories of VEGFb and those of physical activity (SF-IPAQ), which was not significant and did not alter the relationships between the other variables.

**Table 3:** MS development odds ratios according to high levels of serum VEGFb in basal study.

	IDF criteria		ATP-III criteria	
	OR (95%CI)	*p* value	OR (95%CI)	*p* value
Model 1^a^				
High_VEGFb vs Low VEGFb	0.65 (0.43–1.006)	0.053	0.45 (0.27–0.75)	0.002
Model 2^b^				
High_VEGFb vs Low VEGFb	0.61 (0.39–0.96)	0.035	0.38 (0.23–0.65)	<0,001
Model 3^c^				
High_VEGFb vs Low VEGFb	0.65 (0.41–1.02)	0.066	0.41 (0.24–0.70)	0.001
Model 4^d^				
High_VEGFb vs Low VEGFb	0.79 (0.47–1.31)	0.4	0.51 (0.29–0.93)	0.03

^a^Odds ratios calculated by logistic regression adjusted by sex, age and IMC.

^b^Odds ratios calculated by logistic regression adjusted by sex, age, waist circumference, systolic blood pressure, diastolic blood pressure, serum triglycerides, fasting glucose and HDL- cholesterol levels.

^c^Odds ratios calculated by logistic regression adjusted by the same variables than model 2 plus serum hs-CRP and physical activity (IPAQ categories).

^d^Odds ratios calculated by logistic regression adjusted by the same variables than model 3 plus an interaction term between VEGFb levels and physical activity (IPAQ categories).

Additionally, the multivariate analysis for the development of the MS components (Table 4) reported that VEGFb categories were significantly associated with the new onset of abdominal obesity by ATP-III criteria, where subjects with Low_VEGFb category had a 7-fold increased probability of developing abdominal obesity than those with High_VEGFb category; however, when IDF criteria for abdominal obesity diagnosis were considered, the differences did not reach significance. None of the other MS components were significantly associated with the VEGFb categories in any of the tested models.

**Table 4:** Metabolic syndrome components development odds ratios according to high levels of serum VEGFb in basal study.

	High_VEGFb vs Low_VEGFb Minimal adjusted		High_VEGFb vs Low_VEGFb Full adjusted	
	OR(95%CI)	*p*	OR(95%CI)	*p*
Abdominal obesity_IDF^a^	0.71 (0.41–1.25)	0.2	0.70 (0.39–1.25)	0.2
Abdominal obesity_ATP^a^	0.14 (0.03–0.61)	0.009	0.11 (0.02–0.5)	0.005
Hypertriglyceridemia^b^	0.87 (0.52–1.49)	0.6	0.91 (0.52–1.59)	0.7
Low HDL cholesterol^c^	0.691 (0.376–1.269)	0,234	0.65 (0.34–1.24)	0.1
Hypertension^d^	0.899 (0,566–1.427)	0.652	0.91 (0.56–1.45)	0.6
Hyperglicemia^e^	0763 (0.494–1.178)	0.222	1.01 (0.65–1.58)	0.9

^a^Sub-population: Subjects without abdominal obesity by any of the ATPIII or IDF criteria at baseline. Minimal adjusted: Adjusted by age, sex and waist circumference at cross-sectional study.

^b^Sub-population: Subjects without hypertriglyceridemia at baseline. Minimal adjusted: Adjusted by age, sex, and fasting triglycerides levels at cross-sectional study.

^c^Sub-population: Subjects without low HDL cholesterol at baseline. Minimal adjusted: Adjusted by age, sex, and fasting HDL cholesterol levels at cross-sectional study.

^d^Sub-population: Subjects without hypertension at baseline. Minimal adjusted: Adjusted by age, sex, and systolic and diastolic blood pressure at cross-sectional study.

^e^Sub-population: Subjects without hyperglicemia at baseline. Minimal adjusted: Adjusted by age, sex, baseline and fasting glucose levels at cross-sectional study.

*Fully adjusted: additionally adjusted by serum hs-CRP and physical activity (IPAQ categories).

*VEGFb* vascular endothelial growth factor b, *IDF* International Diabetes Federation, *ATP-III* National Cholesterol Education Program Adult Treatment Panel III, *HDL* high-density lipoprotein, *OR* odds ratio, *CI* confidence interval.

## Discussion

In this cohort of the Spanish adult population at risk of developing MS, we found that serum VEGFb levels were inversely associated with the risk of incident MS and abdominal obesity diagnosed by ATP-III, after 7.5 years of follow-up, independently of different risk factors for MS such as age, gender, fasting glucose level, waist circumference, plasma lipids, hypertension, inflammatory state or physical activity.

The metabolic role of VEGFb in humans remains elusive. To date, clinical studies have focused their investigation on comparing VEGFb levels regarding different metabolic disturbances between patients and healthy subjects with discordant results. Some studies have reported increased circulating and adipose tissue VEGFb levels in individuals with obesity compared to lean subjects [26, 33], but the results have not been reproduced by other authors [28]. Sun et al. reported no differences in serum VEGFb levels between subjects with Type 2 Diabetes mellitus (T2DM) and healthy controls [27], however, more recently Wu et al. found elevated plasma VEGFb levels in both individuals newly diagnosed with T2DM and in subjects with impaired glucose regulation compared to normal glucose tolerance subjects [25]. In line with those authors reporting no differences in VEGFb levels according to the presence or not of obesity or T2DM, in our study, BMI, waist circumference, fasting glucose or insulin resistance at baseline study were not different according to VEGFb categories.

Different clinical studies have found a positive correlation between VEGFb levels and circulating triglycerides in patients with T2DM [25, 27]. In line with these investigations, in our study, serum triglyceride levels at baseline were found to be significantly increased in subjects included in the High_VEGFb category compared to those in the lower VEGFb category. Altogether, these results would suggest that VEGFb is associated with circulating triglycerides in humans.

Our results have shown significant higher systolic blood pressure levels at baseline in subjects included in the High_VEGFb category. This result agrees with a previous clinical study reporting a significant correlation between elevated VEGFb plasma levels and blood pressure [23] in individuals with hypertension. Ye et al. have also suggested that plasma VEGFb might be a proper biomarker for the early detection of hypertension in humans [23], but they could not verify this suggestion since their study did not include a follow-up stage. According to our cohort study, serum VEGFb levels were not associated with the new onset of hypertension after 7.5 years.

Our main objective was to investigate the potential association of VEGFb with the future onset of metabolic disturbances related to MS. In this regard, our results have shown that subjects with high VEGFb levels at baseline (over the 75th percentile of the variable), have lower probabilities of developing MS, compared with those with lower VEGFb levels, independently of the age, sex and baseline fasting glucose level, waist circumference, serum lipids, blood pressure, inflammatory state or physical activity level. Moreover, results about the association between VEGFb categories and the development of the individual components of MS have shown that only abdominal obesity incidence by ATP-III criteria is associated with VEGFb levels.

To the best of our knowledge, this is the first report to analyse the association of VEGFb levels along with other variables of interest with the risk of developing MS or its components. Our results in humans might correspond with the observations in animals that VEGFb transgenic overexpression or adipose VEGFb protein delivery alleviate MS, stimulate fat burning and diminish obesity by enhancing the activation of VEGFR2 signaling pathways and thermogenesis [21, 22], effects which are similar to those of physical exercise. This metabolic effect of VEGFb is also in line with other preclinical studies in which adipose and whole body VEGFb knockout mice had a larger volume of white adipose tissues, and reduced metabolic and thermogenic activities than wild type fenotype [34, 35]. Results from these studies suggest that the downregulation of adipose VEGFb may lead to lipid mobilization from brown adipose tissue to be accumulated in white adipose tissue, accompanied by a specific genetic regulation. All these together might explain why in our study subjects with lower VEGFb levels would be more prone to developing abdominal obesity and MS, in comparison to those with higher VEGFb levels. However, these findings would also contrast with other preclinical studies which have proposed that decreased expression or inhibition of VEGFb signaling pathways would prevent excess lipid storage and lead to normalization of glucose levels [19, 20, 36]. As a possible explanation for the coexistence of both apparently opposing behaviors, Zhu et al. compared these animal studies and proposed a context-dependent switch on the VEGFb role [14]. According to Zhu et al. a VEGFb gain-of-function would be applicable when it mainly acts on adipose tissue, while a VEGFb loss-of-therapy would probably occur when it targets on non-adipose tissue. Although it is still unknown how increased adipose VEGFb levels or enhanced adipose perfusion would lead to alleviating or preventing metabolic syndrome in humans, Robciuc et al have proposed that it might be associated with an increased insulin delivery as a consequence of the expansion of the vascular density associated with the effect of VEGFb on the bioavailability of VEGF-A for VEGFR2 [21].

Our results have shown that those subjects with high physical activity presented a low risk of developing MS diagnosed by the ATP-III or IDF criteria, after 7.5 years of follow-up in line with previous literature [37]. We have also evaluated the interrelation of VEGFb levels with other important explanatory variables of MS such as physical activity. In this way, our results have shown that subjects with high VEGFb levels at baseline and high physical activity, presented a lower probability of developing MS by the ATP and IDF criteria compared with those with moderate and low physical activity, but the multivariate analysis did not obtain a significant interaction between both variables, losing the significance of physical activity and maintaining that of VEGFb with the multivariate adjustment (data not shown). These VEGF-physical activity interrelationships might be related with the results of previous animal studies that have reported an increment in the skeletal muscle gene expression levels of different proangiogenic factors, including VEGFb, both in diabetic and healthy mice after exercise training [38, 39]. These authors have suggested that increased angiogenic factors after exercise training may be one of the mechanisms responsible for the beneficial metabolic effects of regular exercise, by stimulating the whole angiogenic signaling pathway and thus, being effective in the prevention and treatment of peripheral vascular problems in metabolic diseases [38, 39]. Another possible mechanism underling the association between VEGFb levels and physical activity in the explanation of the MS development might be related with the fat oxidative capacity of the skeletal muscle. VEGFb produced by skeletal muscle controls the expression of fatty acid (FA) transporter proteins in the capillary endothelium and therefore links endothelial FA uptake to the oxidative capacity of skeletal muscle [19]. Sedentary individuals have lower VEGFB production and are likely to deposit diet fats in ectopic fat depots and develop obesity. As individuals with obesity or sedentary lifestyles have a low capacity for fat oxidation in skeletal muscle, this may also explain the accumulation of diacylglycerols and ceramides in their muscle, which is known to lead to insulin resistance and eventually to type 2 diabetes [40]. However, in lean and non-sedentary people, dietary fats are preferentially oxidized into skeletal muscle and subcutaneous adipose tissues [41]. Altogether, this reinforces the idea that exercise promotes fat oxidation in muscle and white adipose tissue through the activation of fat mobilization systems such as VEGFB, which would contribute to improve the general metabolic status.

The main strength of the study is that the data were obtained from a large national-wide cohort, with a considerable duration of the follow-up and substantial number of events. Most of the participants underwent an OGTT to diagnose diabetes or hyperglycemia. The physical activity of participants was evaluated with an extensively standardized and used test, the SQ-IPAQ. Our study also presents some limitations. Although participation in the follow-up was 66% and the possible participation bias in the whole study was minimal [30], the influence of some confounding factors cannot be ruled out. Additionally, the limited sample size when the population is grouped according to the absence of each individual component of MS might be the cause of the lack of significant associations between VEGFb categories and the risk of developing the different metabolic disturbances. Differences due to interventions carried out in the area of origin of the participants have not specifically been evaluated; nevertheless, all the procedures were performed both at baseline and during follow-up by the same trained nurses, so data and sample collection are expected to have less variability. Finally, the tissue origin or destiny of circulating VEGFb are unknown in this study, so the context-dependent switch hypothesis derived from the preclinical studies could not be tested.

In conclusion, the data gathered from this national cohort demonstrated an independent association between VEGFb levels and the risk of incident MS. Our results indicate that VEGFb may be considered an early indicator of abdominal obesity development in the Spanish adult population without MS. Our results also reinforce the idea that physical activity can be used as a preventive therapy for the pathologies involved in MS perhaps through its possible effect on the VEGFb system. Further investigations with larger sample sizes would be the prerequisite to confirm the association of VEGFb levels with the components of the MS definition, as well as to generalize a risk-score value for predicting incident MS.

## Data Availability

The datasets generated during and/or analysed during the current study are available from the corresponding authors on reasonable request.
